# Cardiorenal syndrome type I recovery following heart rate correction: Cardiac output is not only stroke volume

**DOI:** 10.1002/ccr3.6287

**Published:** 2022-08-26

**Authors:** Ossama Maadarani, Zouheir Bitar, Tamer Zaalouk, Boutros Hanna, Mohamad Elhabibi, Moataz Aldaher, Adnan Hajjia

**Affiliations:** ^1^ Internal Medicine Department/Critical Care Department Ahmadi Hospital—Kuwait Oil Company Al Ahmadi Kuwait; ^2^ Critical Care Department Borg Alarab Central Hospital Alexandria Egypt; ^3^ Clinical Pharmacy Department Ahmadi Hospital—Kuwait Oil Company Al Ahmadi Kuwait

**Keywords:** cardiac output, cardio‐renal syndrome, heart rate, pacemaker

## Abstract

Bradyarrhymias can result in low cardiac output state despite having a normal left ventricular ejection fraction and stroke volume. Because cardiac output is defined as the product of heart rate and stroke volume, a low cardiac output state caused by bradyarrhythmias may result in type I cardiorenal syndrome.

## Graphical abstract

Bradyarrhythmias can result in a low cardiac output state despite having a normal left ventricular ejection fraction and stroke volume. Because cardiac output is defined as the product of heart rate and stroke volume, a low cardiac output state caused by bradyarrhythmias may result in type I cardiorenal syndrome.

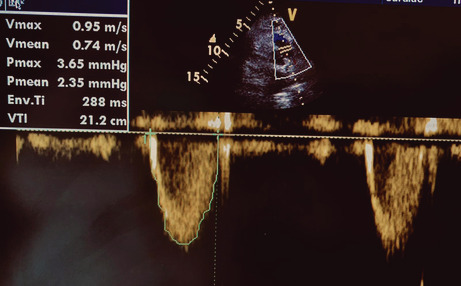

## 1 CASE REPORT

**FIGURE 1:**
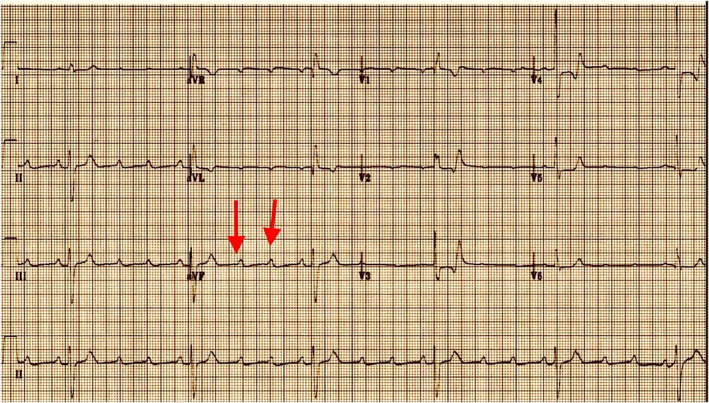
High‐grade Atrioventricular block (AV). Second‐degree heart block with P: QRS ratio of 3:1 or higher, causing extremely slow ventricular rate (33 beat/min). Red arrows denote atrial rate of 140 beat/min

**FIGURE 2:**
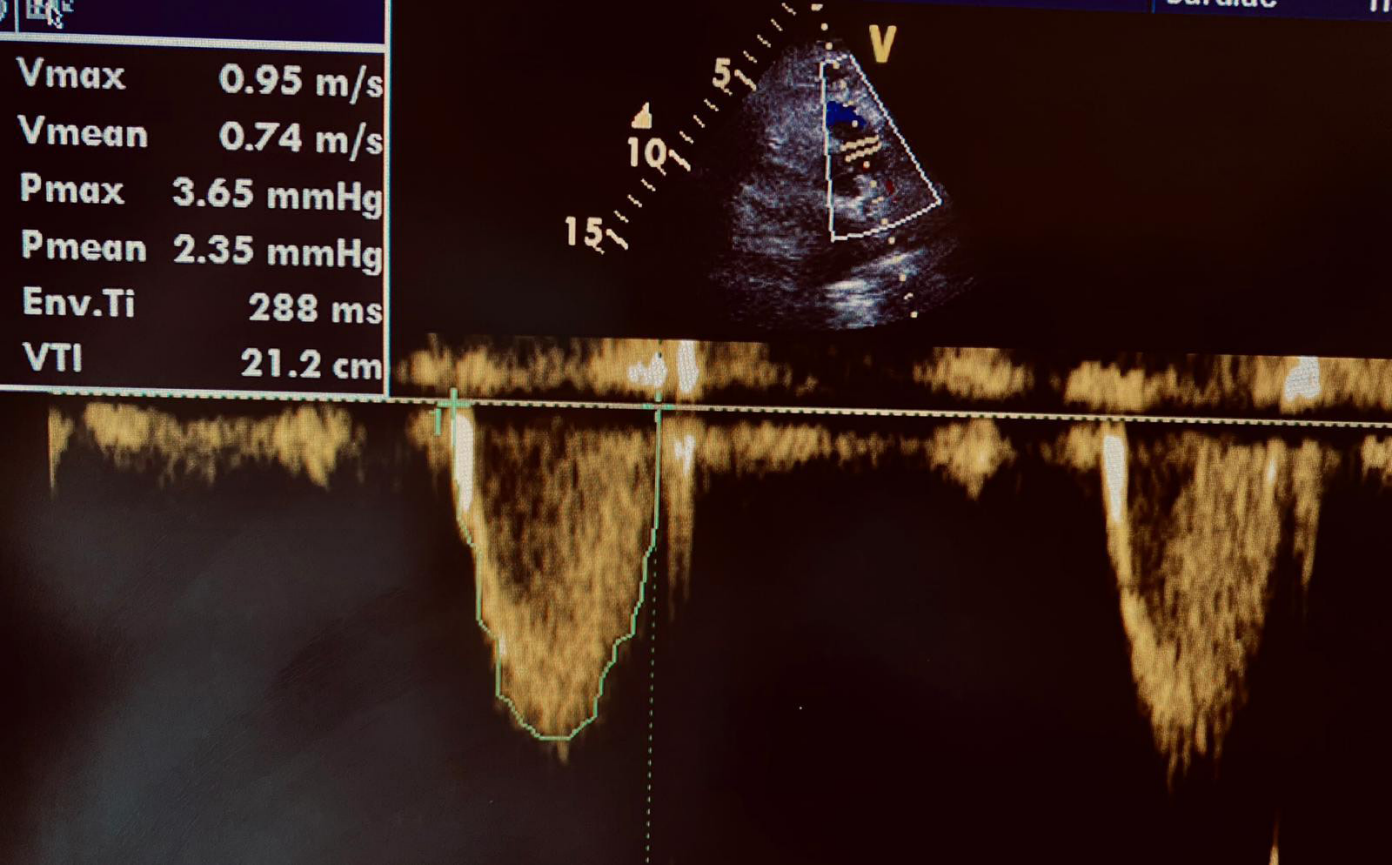
Pulse wave Doppler of left ventricular outflow tract (LVOT). The trace measuring LVOT velocity time integral (VTI)

A 78‐year‐old female patient with a history of hypertension and dyslipidemia presented to the emergency department for a few hours of acute dizziness and severe general fatigue. She denied any chest pain or shortness of breath, and she had previously experienced no such symptoms. She had no prior history of kidney disease, and her kidney function was normal at a routine check‐up a month ago. Moreover, her long‐term medications also included amlodipine 5 mg once daily and atorvastatin 40 mg once daily. Physical examination revealed that the patient was overweight (BMI 29 kg/m^2^), had severe bradycardia of 30 beats per minute at rest, initial blood pressure of 150/80 mmHg, normal respiratory rate, and oxygen saturation. Cardiovascular examination was remarkable for severe bradycardia with no additional heart sounds or murmurs. The rest of the examination was unremarkable. Her electrocardiogram showed a high‐grade atrioventricular (AV) block as a second‐degree heart block with a P: QRS ratio of 3:1 or higher, causing a slow ventricular rate (Figure 1). The patient was admitted to a critical care unit for continuous monitoring of vital signs. Her laboratory results showed a hemoglobin level of 12 g/dl, normal coagulation profile, and normal liver function test. Serum electrolytes were also normal, apart from mild hyperkalemia. Serum lactate was reported as 3 mmol/L. Her kidney function revealed creatinine of 203 μmol/L, blood urea nitrogen (BUN) of 28.5 mmol/L, and albumin of 3.5 g/dl, all of which are new findings as compared with her baseline levels a month ago. Arterial blood gas revealed metabolic acidosis with a pH of 7.32 and HCO3 of 18 mEq/L. Urine analysis showed no evidence of urinary tract infection. Computed tomography of the brain revealed no neurological insult. The echocardiography study revealed the normal left ventricular size and systolic function with an estimated ejection fraction of 65% and grade I diastolic dysfunction. No significant valvular pathology and normal function of the right ventricle were observed. Using velocity time integral (VTI) at the left ventricular outflow tract (LVOT) (Figure 2) and cross‐sectional area (CSA) of LVOT, stroke volume (SV) was calculated at 65 ml per beat, which was considered within the normal range. Due to the low ventricular rate, the cardiac output (CO) was measured at 1.9 L per minute and cardiac index at 1.1 L per minute per meter 2 (L/M/M2), and these values can explain the low cardiac output state.

To improve ventricular rate, the patient received atropine of 1 mg twice with no significant response, and dopamine infusion was initiated and titrated up to 15 mic/kg/min. A measure to decrease potassium level was done and gradually the serum potassium improved and became upper normal value. The response for dopamine infusion was blunted with no improvement in ventricular rate. Continues monitoring of blood pressure showed stable readings without episodes of hypotension. Following a 12‐h follow‐up, renal function revealed increased creatinine, and the patient became anuric while remaining bradycardic. Mixed venous saturation from the central line showed a value of 50%, indicating a state of low CO. Patient parameters did not improve with these therapeutic measures.

**FIGURE 3:**
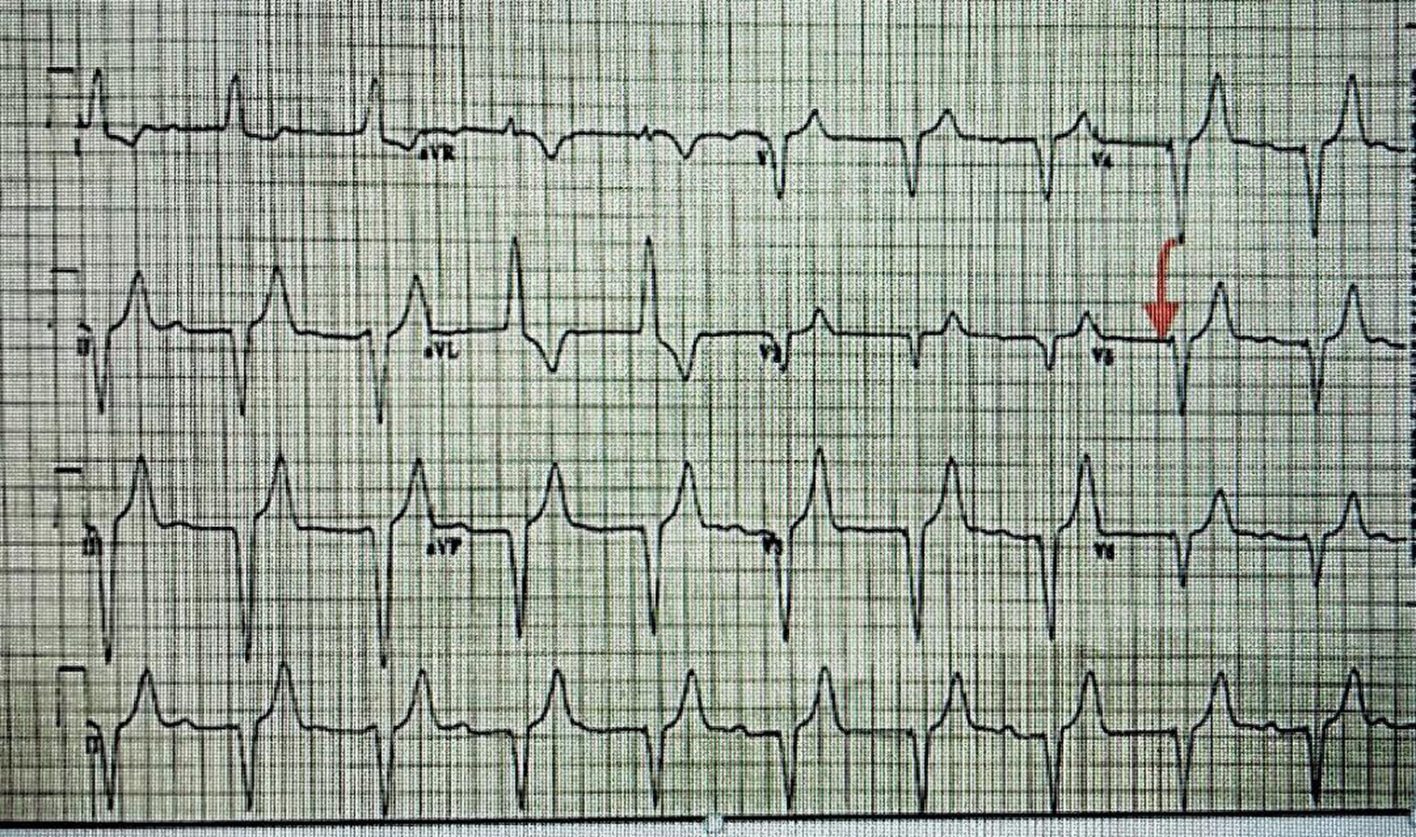
Paced rhythm after transvenous pacemaker insertion. Red arrow—Spike preceded wide QRS complex

The patient was diagnosed with an acute kidney injury and cardiorenal syndrome (CRS) type I due to a low CO state secondary to a low ventricular rate, which was most probably caused by progressive atherosclerotic changes in the atrioventricular node. The possibility of BRASH syndrome was remote since the patient was not on medication blocking the atrioventricular node and had higher normal blood pressure on presentation. To improve heart rate and raise CO, a temporary transvenous single lead pacemaker was inserted with a target heart rate of 60 beats per minute (B/min) (Figure 3) and higher. The output increased from 1.9 to 3.9 L per minute.

Two hours following the insertion of the pacemaker, the symptoms of the patient and urine output improved with the gradual recovery of kidney function. A permanent pacemaker was inserted later on, and the patient was discharged from the hospital with normal kidney function.

In our case, it was essential to recognize early the state of low CO state secondary to bradyarrhythmia with the subsequent manifestation of acute kidney injury and cardiorenal syndrome type I. This early recognition leads to successful treatment with a transvenous pacemaker.

## 2 DISCUSSION

Cardiac output is an essential parameter in the assessment of cardiac function using transthoracic echocardiography. Cardiac output is defined as the product of SV and heart rate. The amount of blood that is pumped out of the ventricles with every beat is called SV. The three variables that determine the SV are preload, contractility, and afterload. Depending on the body's metabolic needs, the range of CO can vary widely. The cardiac index is defined as the CO divided by the body surface area.

Measuring the VTI at LVOT and the CSA of the LVOT^1^ using transthoracic echocardiography (TTE) can determine the SV of the left ventricle. VTI is the distance that blood travels with each beat and can be determined with pulse wave Doppler.^2^ Strove volume is the product of VTI and CSA. Cardiac output is calculated through the following formula: CO = SV × HR.

TTE‐guided measurement of cardiac output has shown a very good correlation with thermodilution‐derived cardiac output measurements,^3^ which is considered the gold standard of CO measurement.

CRS is an umbrella of pathologies of the heart and kidney in which dysfunction of one organ may lead to dysfunction of the other organ and can be in either an acute or chronic fashion.^4^ Cardiovascular comorbidities regularly determine renal function and, vice versa, chronic renal insufficiency accelerates cardiovascular disease. Based on which organ fails first, in 2008, two major categories were identified as cardiorenal and renocardiac syndromes.^5^ According to the Consensus Conference of the Acute Dialysis Quality Initiative, the sequential involvement of organs and the acuity of disease can distinguish five types of CRS. In this classification of CRS, the bidirectional relationship of CRS has been highlighted. CRS types I and II are acute and chronic cardiorenal syndromes, respectively, whereas CRS types III and IV imply acute and chronic renocardiac syndrome, respectively. CRS type five is a secondary CRS to a systemic process leading to heart failure and kidney failure concurrently. Recently an update of the consensus classification of CRS specifies in addition to whether CRS is acute or chronic, whether a valvular or nonvalvular heart disease is present and whether CRS associates with hyper‐ or hypovolemia. Adding these clinical hallmarks (valvular or nonvalvular CRS/hyper‐ or hypovolemic CRS) helps to improve clinical decision and to provide further medical or surgical therapy.^6^

The determinant factors in the pathophysiology of CRS are the hemodynamic crosstalk between organs and the activation of neurohormonal systems (renin–angiotensin–aldosterone axis, sympathetic nervous system, and arginine vasopressin secretion), which will take place as a response to hemodynamic factors.^7^ The venous congestion or reduction in the CO as a result of cardiac dysfunction will lead to a reduction in glomerular filtration rate (GFR) in CRS types I and II.^8^ The venous congestion as a result of increased right ventricular filling pressure in diastolic heart failure impedes the renal venous blood flow and favors an intrarenal edema, which may further impede the intrarenal arterial perfusion leading to acute kidney injury with or without a pre‐existing chronic kidney disease. A renal hypoperfusion during period of cardiac decompensation due to systolic dysfunction or during hypotension will also result in similar consequences.^6^ Type I CRS is a common condition in 25% to 33% of patients admitted with acute decompensated heart failure.^9^ Ronco et al.^10^ described in the literature four subtypes of type I CRS. Subtype 1 of CRS type I indicates new cardiac injury with subsequent new kidney injury, whereas subtype 2 is a new cardiac injury that results in acute‐on‐chronic kidney injury. Subtype 3 occurs when an acute‐on‐chronic cardiac decompensation leads to new kidney injury; subtype 4 is acute‐on‐chronic cardiac decompensation that leads to acute‐on‐chronic kidney injury.^10^ Aoun et al.^11^ reported a case of severe bradycardia as a reversible cause of cardio–renal–cerebral syndrome type I. Pliquett et al.^12^ demonstrated that bradycardia and pre‐existing low diastolic kidney perfusion were showed to be the cause for oliguric acute kidney injury stage 3. Other authors suggested to include bradyarrhythmias in the cardiorenal syndrome etiology as a cause of low CO in Type I CRS which can be successfully treated with pacing.

Our case demonstrated a subtype 1 of CRS type I when a bradyarrhythmia resulted in de novo cardiac injury and a low CO state, leading to acute kidney injury. Early recognition of the role of severe bradyarrhythmia in a low CO state using TTE resulted in early correction of a reversible cause and subsequent recovery of kidney function.

## 3 CONCLUSION

Cardiac output is not only SV. Severe bradyarrhythmia can be a cause of low CO state and lead to cardiorenal syndrome type I which can be successfully treated with pacing.

## AUTHOR CONTRIBUTIONS

OM wrote the article, ZB and TZ shared in the discussion, and MA, BH, MA, and AH collected the data and revision of the manuscript. Our working website is www.kockw.com (Kuwait Oil Company, Ahamdi Hospital).

## FUNDING INFORMATION

None.

## CONFLICT OF INTEREST

The author(s) declare no potential conflicts of interest with respect to the research, authorship, and/or publication of this article.

## CONSENT

Informed consent was obtained from the patient for the publication of this case report.

## Data Availability

The data that support the findings of this study are available from the corresponding author upon reasonable request.
